# Challenges faced by acute care surgeons in China

**DOI:** 10.1186/s13017-019-0236-3

**Published:** 2019-04-02

**Authors:** Dequan Xu, Limin Hou, Haoxin Zhou

**Affiliations:** Department of Emergency Surgery, First Affiliated Hospital of Harbin Medical University, 23 Youzheng Street, Nangang District, Harbin, 150001 Heilongjiang Province People’s Republic of China

**Keywords:** ACS, Elderly patients, ED visits, EGS

## Abstract

The aim of this article is to describe briefly about Chinese ACS surgeons’ work status. It is an undeniable fact that the analysis of ED and ACS resources shows negative tendencies and high work overload, resulting in low patient safety and quality of care. And, there was a substantial shortage of surgeons in the subspecialty. So, a set of strategic measures and state policies should be prioritized.

## Background

**Fig. 1:**
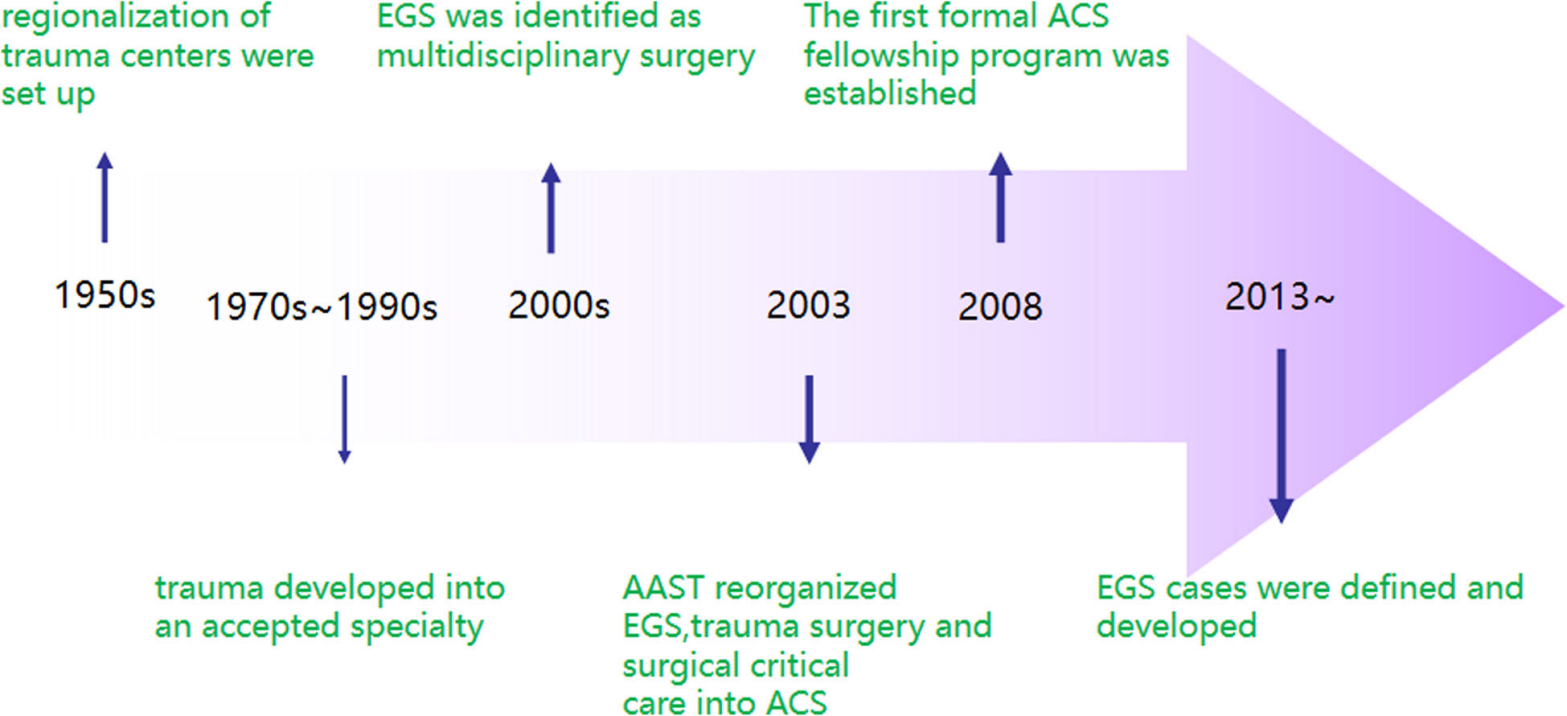
Development process of ACS. EGS emergency general surgery, AAST the American Association for the Surgery of Trauma

**Fig. 2:**
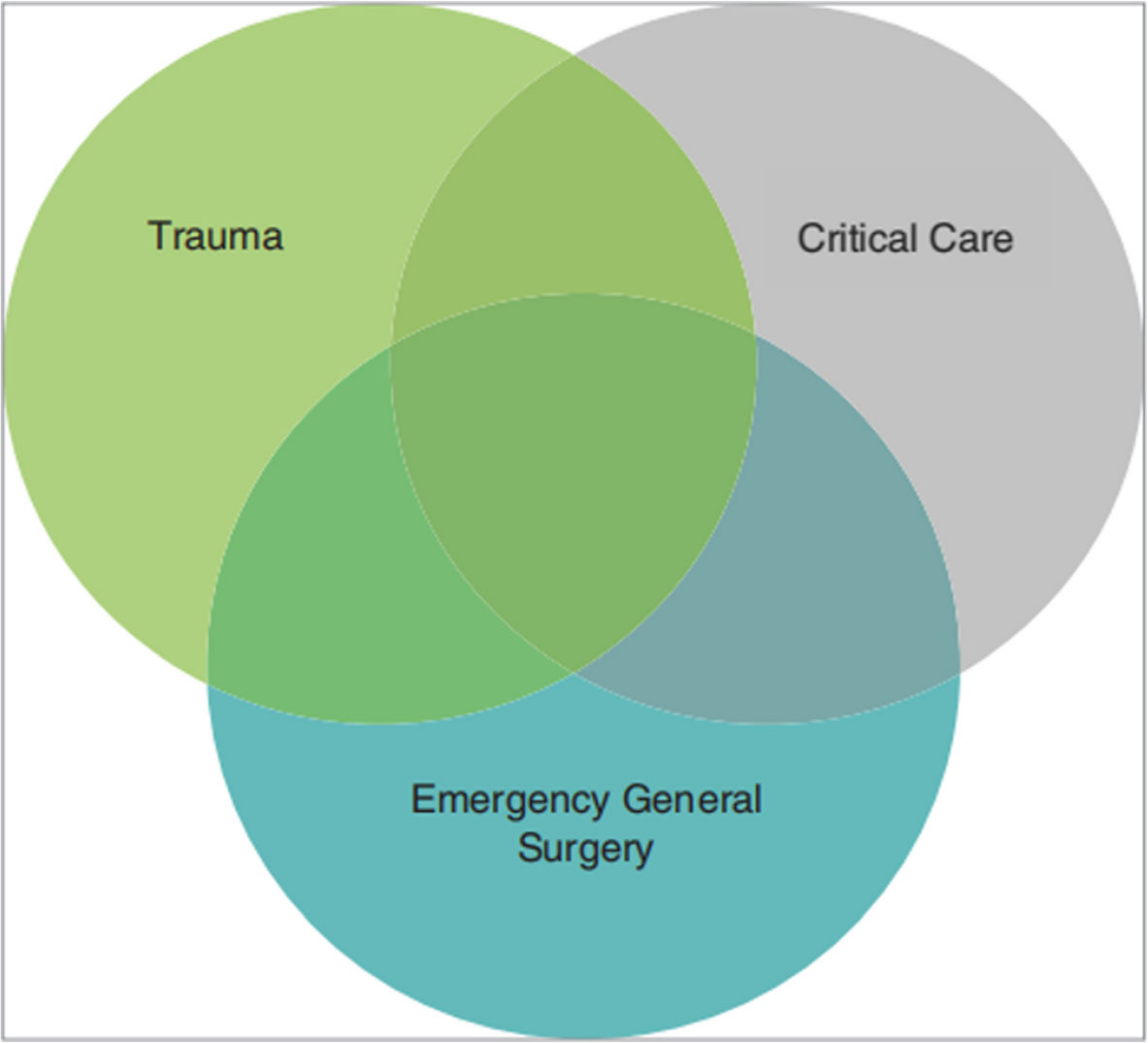
ACS: a tri-disciplinary specialty

Acute care surgery (ACS) as a distinct subspecialty within general surgical practice has been in existence for over a decade [1, 2] (Fig. 1). Although often used interchangeably, “emergency general surgery (EGS)” and “ACS” have different meanings. Whereas EGS refers to acute general surgical disorders, ACS includes surgical critical care and the surgical management of acutely ill patients with a variety of conditions including trauma, burns, surgical critical care, or an acute general surgical condition [3] (Fig. 2). Previous studies have demonstrated the improvement in injury-related mortality and length of stay in hospital systems that use ACS model [4, 5].

China’s rapid economic growth and resulting demands for modern health care have resulted in heavy pressure on ACS surgeons. Chinese ACS surgeons are overworked, suffer lack of respect, and faced the possibility of serious employee turnover. As a consequence, they are faced with some dilemmas in China, which need to be settled urgently.

## Main text

### Concerns from population growth

By the end of 2016, China’s population approached 1.38 billion. Despite its rapid economic development, China has not seen its health care system keep pace. In 2016, China had 2.31 licensed (assistant) doctors per 1000 population, which was substantially lower than other countries.

**Fig. 3:**
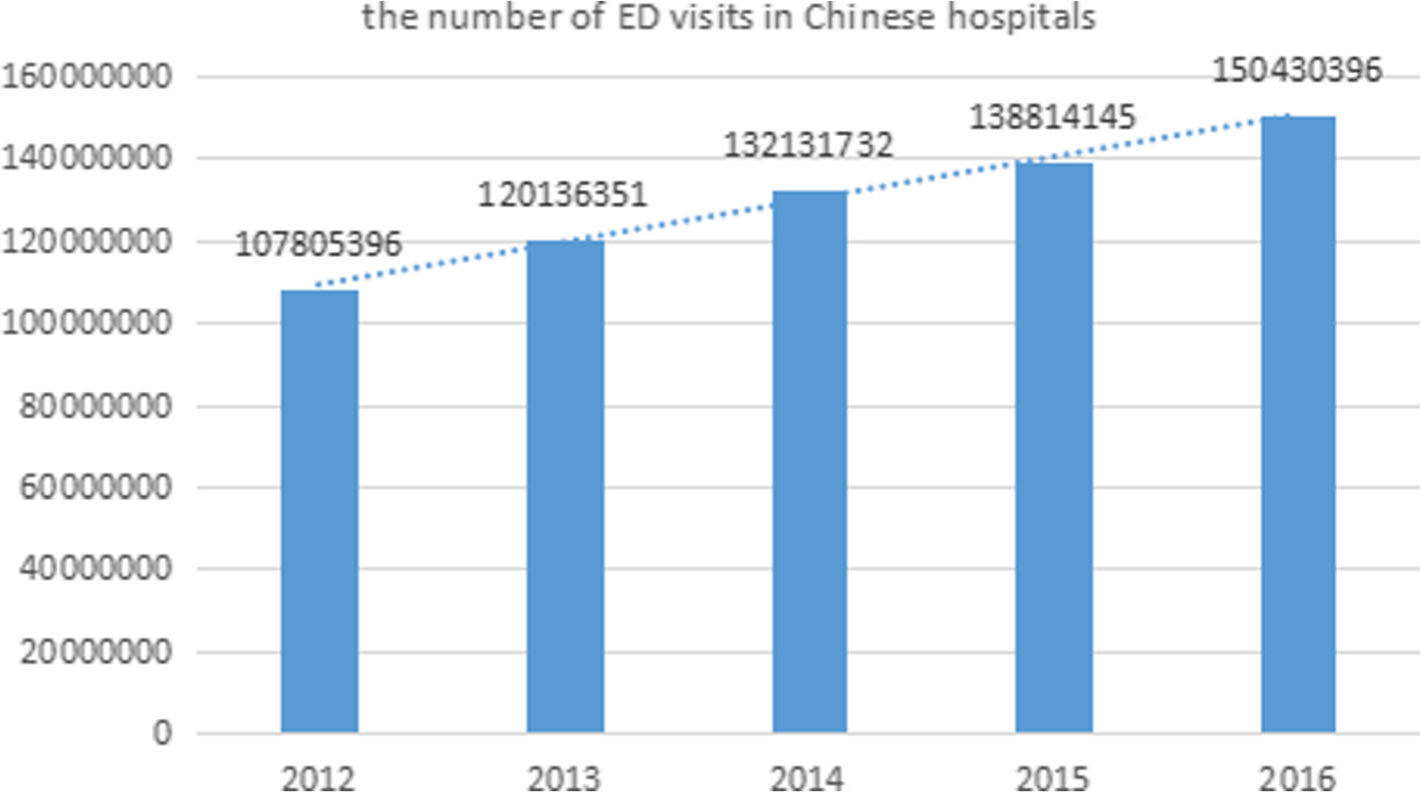
The number of ED visits in Chinese hospitals during the years 2012–2016

In China, hospitals have noted an increased acuity of patients presenting to them. According to the “2017 China Health Statistical Yearbook Compiled by the National Health and Family Planning Commission (http://www.yearbookchina.com/),” the number of outpatients in hospitals has increased significantly from 2,483,091,057 in 2012 to 3,197,103,337 in 2016 over a 5-year period. The population of medical practitioners, outpatients, and emergency department (ED) visits in Chinese hospitals during the years 2012–2016 is shown in Table 1. From the present statistical data, the population of medical practitioners, outpatients, and ED visits had grown over 29.5%, 28.8%, and 39.5% by 2016 relative to the 2012 baseline, respectively. The ED visit population grew at a faster rate than the previous two. The trend in the number of ED visits in Chinese hospitals from 2012 to 2016 is shown in Fig. 3.

**Table 1:** Population of medical practitioners, outpatients, and ED visits in Chinese hospitals during the years 2012–2016

Years	Medical practitioners	Outpatients	ED visits
2012	1,297,078	2,483,091,057	107,805,396
2013	1,392,732	2,679,015,728	120,136,351
2014	1,470,470	2,902,939,060	132,131,732
2015	1,573,093	3,016,549,832	138,814,145
2016	1,680,062	3,197,103,337	150,430,396

Unavoidable incidents, which may refer to unintentional injuries due to falls, motor vehicle traffic crashes, fire/hot objects, or other external causes, represent a large proportion of ED visits. There is no doubt that ACS surgeons are under the burden of a heavy workload that comes with exploding ED visits. However, higher workload for emergency physicians can negatively impact patient safety and quality of care. Safety and quality improvement is of particular importance in the field of ACS and has been required more attention with increased ED visits. As demand continues to grow, workloads increase, and treatments become more complex, a critical need for a departmental and institutional initiative in safety and quality control is apparent.

### Concerns from elderly patients

**Fig. 4:**
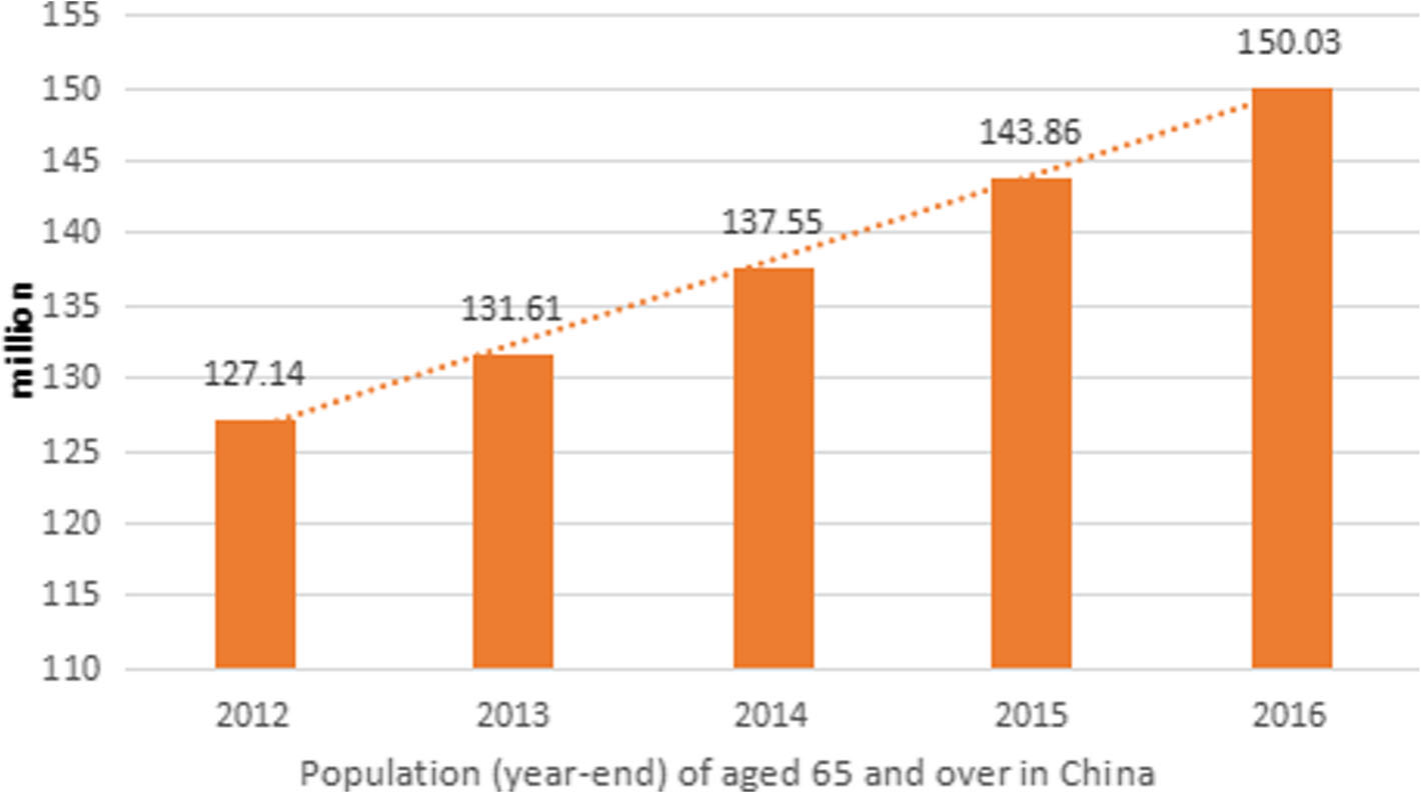
Population (year-end) of aged 65 and over in China

China had entered an aging society by the end of the last century. As age increases, health status and medical demands change correspondingly. According to the “2017 China Statistical Yearbook Compiled by National Bureau of Statistics of China (http://www.stats.gov.cn/),” people aged 65 and over accounted for 10.8% (150.03 million) of the total population in 2016 in China; by 2030, the population is predicted to be 1.46 billion, and 16% percent of Chinese citizens will be aged 65 and over. Figure 4 shows that the prevalence of the population aged 65 and over has increased significantly from 124.14 million in 2012 to 150.03 million in 2016 over a 5-year period in China.

The population is aging, with octogenarians representing the most rapidly growing segment, and the resources required for the elderly with acute severe illnesses are greater than for younger patients with the same emergencies. Some Chinese research has shown that emergency visits per capita in the elderly group (aged 60 and over) was 4.1 and 4.5 times higher than the childhood group (aged 1–14) and the youth and adult group (aged 15–59); hospitalization per capita in the elderly group was 3.0 and 3.5 times higher than the childhood group and the youth and adult group [6]. Presenting with acute surgical disease, elderly patients tend to have considerably more co-morbidities and active medical problems. Some research has shown that an aging Chinese population will lead to steep increases in the number of CHD events and deaths in persons ≥ 65 years old [7]. With the preexisting conditions, or comorbidities, increasing with age, the finding by Schloss [8] that the elderly require twice the time and effort of the general surgeon is even more relevant for the elderly patient in the acute care setting. All of these factors speak to the urgent need for a system designed to coordinate and ensure access to emergency surgical care.

### Concerns from job satisfaction

Job satisfaction is identified as an evaluative state that expresses contentment with and positive feeling about one’s job. Physician job satisfaction may be affected by job demands, job control, doctor-patient relationship, income, and incentives. It is regrettable that most acute care surgeons have low job satisfaction due to not only job demands but also doctor-patient relationship and income.

The working environments for doctors have severely deteriorated in the past 20 years [9]. Violence against doctors has been reported repeatedly and such incident was more common in the emergency department. Furthermore, numerous studies indicate that the doctor-patient relationship in China is facing serious challenges, and mass media sometimes has not played a positive role in harmonizing the relationship [10]. What is more, it is well known that the complication or mortality rate of emergency surgery is higher than that of elective surgery. However, ACS surgeons still are subject to fines or imprisonment in the event of treatment failure or complications. All doctors in China work without medical liability insurance and they have to pay these fines from their personal income.

It is a historical irony that today, there are well-trained and qualified ACS surgeons committed to providing emergency department coverage 24 h a day, but challenged to maintain salary targets consistent with other specialty fields solely based on a traditional relative reimbursement structure. This situation is basically the same at home and abroad. Previous studies have demonstrated that yearly ACS salary would need to be increased by 28% to yield the mean specialty net present value (NPV) and to produce financial neutrality in decision-making [11].

### Concerns from discipline development

Standardized training of residents plays an important role in the growth process of young ACS surgeon. In 2005, the American Association for the Surgery of Trauma (AAST) developed and implemented the Acute Care Surgery Fellowship Training program for the training of surgeons in ACS. However, emergency medicine and critical care medicine are the new contents in standardized training of residency in China. Although curriculums for standardized resident education continue to evolve in China, there is no standardized model for ACS surgeons. The system of standardized training residency lacks for a traditional way to refer.

What should be of concern is that a large number of Chinese medical students choose to get away from ACS. It is becoming increasingly difficult for ACS training programs to attract the best qualified medical students, and there is cause for concern about the future of this specialty. Several studies have shown that a stable, predictable lifestyle is a very important factor in medical students’ selection of a specialty and that this desire for a controllable lifestyle is the key factor contributing to the reduction in the number of graduates who choose fields such as ACS [12, 13]. Moreover, the general surgery workforce has followed a trend of increased specialization, with young surgeons gravitating toward specialties that are perceived to have a better development, which especially when compared with the ACS, has had no prominent place in most European countries, with no formal ACS specialists in most countries.

## Conclusions

There was a fast growth in the number and the formation of the elderly in China during the twenty-first century. The proportion of the elderly in the total population will increase gradually. In this environment, increasing demands for ACS surgeons, we sought to determine the current state of the workforce in ACS. It is easy to see from our research that the analysis of ED and ACS resources show negative tendencies and high work overload, resulting in low patient safety and quality of care, requiring structural changes. Meanwhile, adequate and precise assessment of job dissatisfaction from practicing ACS surgery discloses the negative concerns about the discipline development. We hypothesized that there was a substantial shortage of surgeons in the subspecialty. In addition, we think that ACS surgeons’ urgent problem to be solved is mainly to impel ACS model construction, improve ACS surgeons’ salary, formulate career promotion system, and protect their quality by the government.

## Abbreviations

AAST: The American Association for the Surgery of Trauma
ACS: Acute care surgery
ED: Emergency department
EGS: Emergency general surgery
NPV: Net present value
